# A genome-wide association study suggests the HLA Class II region as the major susceptibility locus for IgA vasculitis

**DOI:** 10.1038/s41598-017-03915-2

**Published:** 2017-07-11

**Authors:** Raquel López-Mejías, F. David Carmona, Santos Castañeda, Fernanda Genre, Sara Remuzgo-Martínez, Belén Sevilla-Perez, Norberto Ortego-Centeno, Javier Llorca, Begoña Ubilla, Verónica Mijares, Trinitario Pina, José A. Miranda-Filloy, Antonio Navas Parejo, Diego de Argila, Maximiliano Aragües, Esteban Rubio, Manuel León Luque, Juan María Blanco-Madrigal, Eva Galíndez-Aguirregoikoa, David Jayne, Ricardo Blanco, Javier Martín, Miguel A. González-Gay

**Affiliations:** 1 Epidemiology, Genetics and Atherosclerosis Research Group on Systemic Inflammatory Diseases, Rheumatology Division, Hospital Universitario Marqués de Valdecilla, IDIVAL, Santander, Spain; 2 Instituto de Parasitología y Biomedicina ‘López-Neyra’, CSIC, PTS Granada, Granada, Spain; 3 Departamento de Genética e Instituto de Biotecnología, Universidad de Granada, Granada, Spain; 4 Rheumatology Department, Hospital Universitario La Princesa, IIS-IPrincesa, Madrid, Spain; 5 Medicine Department, Hospital Universitario San Cecilio, Granada, Spain; 6 Epidemiology and Computational Biology Department, School of Medicine, University of Cantabria, and CIBER Epidemiología y Salud Pública (CIBERESP), IDIVAL, Santander, Spain; 7 Division of Rheumatology, Hospital Universitario Lucus Augusti, Lugo, Spain; 8 Nephrology Department, Hospital Universitario San Cecilio, Granada, Spain; 9 Dermatology Department, Hospital Universitario La Princesa, IIS-IPrincesa, Madrid, Spain; 10 Rheumatology Department, Hospital Universitario Virgen del Rocío, Sevilla, Spain; 11 Rheumatology Department, Hospital Universitario de Basurto, Bilbao, Spain; 12 Department of Medicine, University of Cambridge, Cambridge, UK; 13 School of Medicine, University of Cantabria, Santander, Spain; 14 Cardiovascular Pathophysiology and Genomics Research Unit, School of Physiology, Faculty of Health Sciences, University of the Witwatersrand, Johannesburg, South Africa

## Abstract

The genetic component of Immunoglobulin-A (IgA) vasculitis is still far to be elucidated. To increase the current knowledge on the genetic component of this vasculitis we performed the first genome-wide association study (GWAS) on this condition. 308 IgA vasculitis patients and 1,018 healthy controls from Spain were genotyped by Illumina HumanCore BeadChips. Imputation of GWAS data was performed using the 1000 Genomes Project Phase III dataset as reference panel. After quality control filters and GWAS imputation, 285 patients and 1,006 controls remained in the datasets and were included in further analysis. Additionally, the human leukocyte antigen (HLA) region was comprehensively studied by imputing classical alleles and polymorphic amino acid positions. A linkage disequilibrium block of polymorphisms located in the HLA class II region surpassed the genome-wide level of significance (OR = 0.56, 95% CI = 0.46–0.68). Although no polymorphic amino acid positions were associated at the genome-wide level of significance, P-values of potential relevance were observed for the positions 13 and 11 of HLA-DRB1 (P = 6.67E-05, P = 1.88E-05, respectively). Outside the HLA, potential associations were detected, but none of them were close to the statistical significance. In conclusion, our study suggests that IgA vasculitis is an archetypal HLA class II disease.

## Introduction

Immunoglobulin-A (IgA) vasculitis, also known as Henoch-Schöenlein purpura (HSP), is the most common type of primary small-sized blood vessel leukocytoclastic vasculitis in children, although it may also develop in adults^1^. Although the classic clinical triad of IgA vasculitis consists of palpable purpura (involving the lower extremities), joints and the gastrointestinal tract, renal complications may also develop in affected individuals^2^. In this regard, the outcome of IgA vasculitis patients is related to the presence of glomerulonephritis, which may lead to chronic renal failure^1, 2^.

IgA vasculitis has a multifactorial etiology in which both environmental and genetic factors seem to contribute to the predisposition and clinical phenotype of the disease^1, 3^. However, the genetic component of this type of vasculitis remains poorly understood, as only a few candidate gene studies have been performed to date^4, 5^.

Unlike the candidate gene approach, genome-wide association studies (GWAS) imply a hypothesis free analysis of hundreds of thousands of single-nucleotide polymorphisms (SNPs) across the whole genome^6^. This strategy has proven to be a powerful tool to unravel the genetic component of complex diseases during the last decade, including primary vasculitides such as Takayasu Arteritis, Behçet disease, and antineutrophil cytoplasmic antibody (ANCA)-associated vasculitis (AAV)^7^.

This study aimed at conducting the first GWAS of IgA vasculitis using the largest series of IgA vasculitis patients of European ancestry ever assessed for a genetic study.

## Patients and Methods

### Study population

A series of 308 patients diagnosed with IgA vasculitis and 1,018 unaffected and unrelated controls were genotyped in this study. The total number of individuals that passed the quality control (QC) filters mentioned below and were finally included in further analysis was 1,291 (285 and 1,006 for IgA vasculitis patients and controls, respectively). All subjects were from Spain and had European ancestry. IgA vasculitis condition was diagnosed accordingly with both the guidelines included in Michel *et al*.^8^ and the American College of Rheumatology classification criteria for this form of vasculitis^9^. A description of the main clinical features of the IgA vasculitis patients and controls analyzed after QC filters is shown in Supplementary Table S1. For experiments involving humans and the use of human blood samples, all the methods were carried out in accordance with the approved guidelines and regulations, according to the Declaration of Helsinki. All experimental protocols were approved by the Ethics Committees of clinical research of the Spanish regions of Galicia, Cantabria, Madrid, Andalucía, and País Vasco. All participants or their parents signed an informed consent form before being enrolled in the study.

### Genotyping and quality controls

Genomic DNA was extracted from peripheral blood samples using standard methods. Genotyping was conducted using the GWAS platform “Infinium® HumanCore Beadchip” in an iScan System (Illumina, Inc) and following the manufacturer’s protocol.

Raw data were subjected to the following QC filters using PLINK v.1.07^10^: (1) SNPs with cluster separation <0.4, call rates <0.98, minor allele frequencies (MAF) <0.01, and those deviating from Hardy-Weinberg equilibrium (HWE; P < 0.001) were excluded; (2) samples with call rates <0.95, and those with identity by descent >0.4 were also removed. Sex chromosomes were not analyzed. The number of IgA vasculitis patients and controls that remained after each QC filter is shown in Supplementary Table S2.

### Imputation of GWAS data

SNP genotype imputation throughout the genome was performed after initial QC using the 1000 Genomes Project (1KG) Phase III dataset as reference panel (www.1000genomes.org) and the software IMPUTE v.2^11^. For that, we set the strand orientation, chromosome position, and SNP nomenclature accordingly with the build 37 (HG19) of the 1KG using PLNK. Imputation was carried out in individual chunks of 50,000 Mb covering whole-genome regions with a probability threshold for merging genotypes of 0.9 to maximize the quality of imputed variants. Imputed data were also subjected to the above mentioned QC filters in PLINK. Singletons were removed. Finally, possible population sub-stratification was controlled by principal component (PC) analyses using PLINK and the gcta64 and R-base software under GNU Public license v2. To identify outliers, we calculated and plotted the ten first PCs of each individual, and those deviating >4 standard deviations from the cluster centroid were excluded. PC analysis for the first three PCs for each individual are plotted in Supplementary Fig. The total number of SNPs that passed the QC and were finally analyzed was 1,909,910 (2,581,927 and 2,185,351 for IgA vasculitis patients and controls, respectively). The number of polymorphisms that remained after each QC filter is shown in Supplementary Table S2.

### Human leukocyte antigen (HLA) imputation

Considering that IgA vasculitis is an immune-mediated condition, a more comprehensive analysis of the HLA region was conducted. With that aim, we extracted the extended HLA region (29,000,000 to 34,000,000 bp in chromosome 6) from the non-imputed data and imputed SNPs, classical HLA alleles at two- and four-digits, and polymorphic amino acid positions as described^12–16^. In brief, to impute this genomic region, we used the SNP2HLA method with the Beagle software package and the Type 1 Diabetes Genetics Consortium (T1DGC) reference panel comprised of 5,225 individuals of European origin with genotyping data of 8,961 common SNPs and indel polymorphisms across the xMHC region, and four digits genotyping data of the HLA class I and II molecules^12–16^. Imputed HLA data were also filtered with PLINK with the following thresholds: success call rate >0.95 for alleles and amino acids, deviation from HWE (P < 0.001) for SNPs, and >0.95 total call rate for individuals. Information of a total of 7,179 SNPs, 423 classical HLA alleles (126 at two-digit and 297 at four-digit resolution) of the *HLA-A, HLA-B, HLA-C, HLA-DRB1, HLA-DQB1, HLA-DQA1, HLA-DPB1* and *HLA-DPA1* genes, and 1,275 amino acidic variants of the HLA system remained after the filters.

### Statistical analyses

An estimation of the statistical power of the final cohort (285 IgA vasculitis patients/1,006 healthy controls) was obtained with CaTS Power Calculator for Genetic Studies software (Supplementary Table S3).

To test for association, we compared the genotype frequencies of every SNP between cases and controls by logistic regression on the best-guess genotypes assuming an additive model in PLINK. The ten first PCs were included as covariates. In the case of the HLA region, we tested SNPs, classical HLA alleles and all possible combinations of amino acid residues per position. A likelihood ratio test of amino acid positions was also conducted, as described^12^.

P-values, odds ratios (OR), and 95% confidence intervals (CI) were then calculated. The statistical threshold was set at the genome-wide level of significance (P < 5E-08). In the HLA analysis, despite not interrogating the whole genome but a specific region of chromosome 6, we decided to maintain the statistical threshold at the genome-wide level of significance (P < 5E-08) to avoid possible false positive results.

## Results

**Figure 1:**
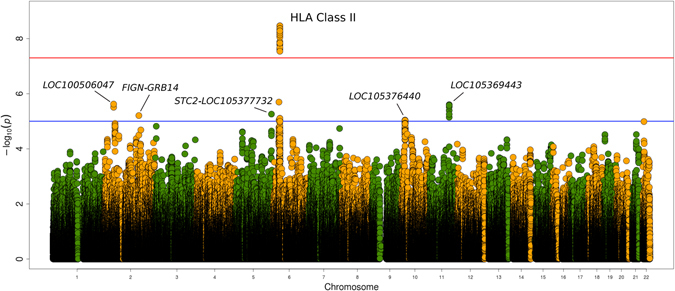
Manhattan plot representation of the results of this study. The −log10 of the p values are plotted against their physical chromosomal position. The red line represents the genome-wide level of significance (P < 5E-08). A less stringent threshold (p < 1E-05) is highlighted in blue.

Figure 1 summarizes the overall results of the study. Several association signals in high linkage disequilibrium (LD, r^2^ > 0.8) at the genome-wide level of significance were disclosed within the HLA region at chromosome 6. The strongest signal corresponded to a disequilibrium block of polymorphisms (OR = 0.56, 95% CI = 0.46–0.68) (Table 1), which we refer to as rs9275260, that maps to an intergenic region in HLA class II between *HLA-DQA1* and *HLA-DQB1*. To confirm these results, we obtained direct genotypes of the Spanish cohort using a TaqMan probe for rs9275260. The overall concordance reached after comparing TaqMan types with the corresponding imputed data was 99.84%. Outside the HLA, some potential signals located in different intronic and intergenic regions were observed (Fig. 1), but none of them reached the statistical level of significance (Supplementary Table S4).

**Table 1:** Signals within HLA associated with IgA susceptibility at the GWAS significance level-P < 5E-08-after imputation of GWAS data.

SNP	Position in chr 6 (GRCh37)	Reference allele	P	OR [CI 95%]
rs9275260	32.661.575	C	3.42E-09	0.56 [0.46–0.68]
rs9275259	32.661.572	C	3.42E-09	0.56 [0.46–0.68]
rs9275284	32.663.073	C	4.30E-09	0.56 [0.46–0.68]
rs9275285	32.663.080	A	4.30E-09	0.56 [0.46–0.68]
rs9275286	32.663.143	T	4.30E-09	0.56 [0.46–0.68]
rs9275288	32.663.203	A	4.30E-09	0.56 [0.46–0.68]
rs9275292	32.663.289	C	4.30E-09	0.56 [0.46–0.68]
rs9275244	32.660.881	G	4.92E-09	0.56 [0.46–0.68]
rs5000633	32.663.610	C	5.20E-09	0.56 [0.46–0.68]
rs2395522	32.664.722	A	5.25E-09	0.56 [0.46–0.68]
rs9275279	32.662.843	G	5.32E-09	0.56 [0.46–0.68]
rs9275281	32.662.920	G	5.32E-09	0.56 [0.46–0.68]
rs4248168	32.659.743	G	5.46E-09	0.56 [0.47–0.68]
rs9275224	32.659.878	A	5.46E-09	0.56 [0.47–0.68]
rs4713580	32.659.994	C	5.46E-09	0.56 [0.47–0.68]
rs4713584	32.660.237	C	5.46E-09	0.56 [0.47–0.68]
rs9275225	32.660.262	G	5.46E-09	0.56 [0.47–0.68]
rs5002704	32.659.279	T	5.67E-09	0.56 [0.46–0.68]
rs4713581	32.660.023	T	6.08E-09	0.56 [0.47–0.68]
rs4713583	32.660.153	T	6.08E-09	0.56 [0.47–0.68]
rs9275228	32.660.347	G	6.12E-09	0.56 [0.47–0.68]
rs9275227	32.660.337	C	6.12E-09	0.56 [0.47–0.68]
rs9275295	32.663.391	A	6.19E-09	0.56 [0.46–0.68]
rs9275277	32.662.677	G	7.33E-09	0.57 [0.47–0.69]
rs9275276	32.662.676	T	7.33E-09	0.57 [0.47–0.69]
rs9275245	32.660.943	A	7.89E-09	0.57 [0.47–0.69]
rs5002708	32.659.357	T	8.00E-09	0.57 [0.47–0.69]
rs5002707	32.659.337	T	8.00E-09	0.57 [0.47–0.69]
rs67838634	32.662.128	G	8.89E-09	1.76 [1.45–2.13]
rs9275222	32.659.516	T	1.28E-08	1.75 [1.44–2.12]
rs6457617	32.663.851	C	1.44E-08	0.57 [0.47–0.70]
rs6457620	32.663.999	G	1.44E-08	0.57 [0.47–0.70]
rs5002702	32.659.158	G	1.47E-08	0.57 [0.47–0.70]
rs9275226	32.660.311	C	1.49E-08	0.57 [0.47–0.70]
rs9275230	32.660.442	A	1.49E-08	0.57 [0.47–0.70]
rs5002705	32.659.319	C	1.60E-08	0.57 [0.47–0.70]
rs4713582	32.660.051	T	1.63E-08	0.57 [0.47–0.70]
rs4711304	32.660.170	T	1.65E-08	0.58 [0.47–0.70]
rs9275246	32.661.003	C	2.11E-08	0.58 [0.48–0.70]
rs4713587	32.659.535	G	2.36E-08	0.58 [0.48–0.70]
rs9275247	32.661.015	T	2.87E-08	0.58 [0.48–0.70]
rs9275231	32.660.505	C	2.81E-08	1.73 [1.42–2.09]

HLA: Human leukocyte antigen; IgA: Immunoglobulin-A; GWAS: genome-wide association study; SNP: single nucleotide ﻿polymorphism; chr: chromosome; OR: odds ratio; CI: confidence interval.

**Figure 2:**
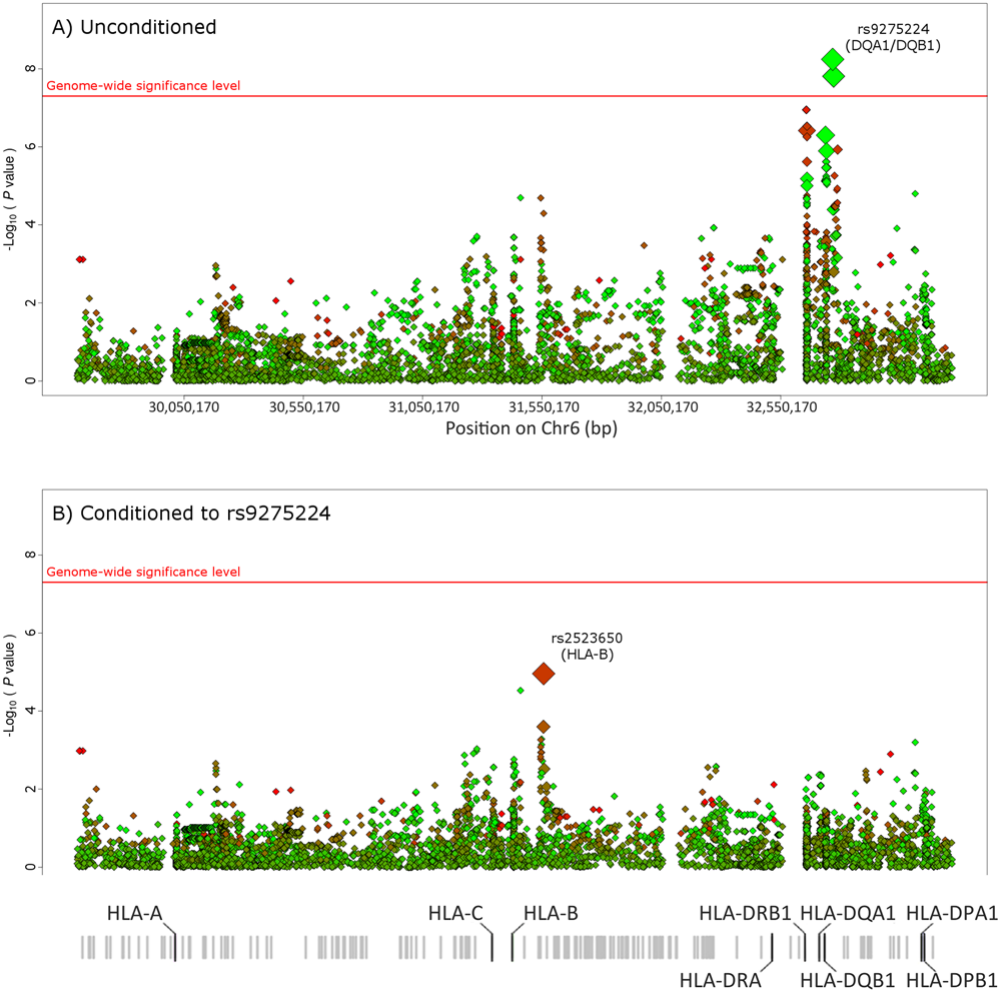
Manhattan plot representation of the step-wise conditional logistic regression of the HLA region. (**A**) Unconditioned test of the HLA region. (**B**) Results of the HLA region after controlling for rs9275224. The −log10 of the p values are plotted against their physical chromosomal position. A red/green color gradient was used to represent the effect size of each analyzed variant (red for risk and green for protection). The red line represents the genome-wide level of significance (P < 5E-08).

We tried to narrow down the HLA association with IgA vasculitis by inferring SNPs, classical HLA alleles, and polymorphic amino acid positions using as reference the T1DGC panel. Accordingly, association signals at the genome-wide level of significance were disclosed (Fig. 2A). The genetic variant rs9275224 represented the strongest peak (P = 5.74E-09, OR = 0.56, 95% CI = 0.46–0.68) (Supplementary Table S5). The polymorphism rs9275260 (and SNPs in high linkage disequilibrium with it) observed in the genome-wide data analysis was not detected in the analysis of the HLA region since 1KG Phase III dataset was not used as reference panel in this analysis. Nevertheless, rs9275224 was in complete LD (r^2^ = 1) with rs9275260 (and, consequently, with all the SNPs of the same disequilibrium block) observed in the genome-wide data analysis, meaning that these polymorphisms represent the same signal. Although no polymorphic amino acid positions were associated at the genome-wide significance level, P-values of potential relevance were observed for the HLA-DRB1 positions 13 and 11 (P = 6.67E-05 and P = 1.88E-05, respectively) (Supplementary Table S6). Conditional logistic regression analyses of the HLA data indicated that rs9275224 explained most of the HLA associated variants in HLA class II (Fig. 2B). Regarding HLA class I, a potential signal in *HLA-B* was observed (rs2523650, P = 1.10E-05, OR = 1.59, 95% CI = 1.29–1.96).

## Discussion

This study represents the first GWAS of IgA vasculitis. Consistent with the results obtained in a former study^5^, our data suggest the involvement of HLA class II region in the pathophysiology of IgA vasculitis, thus supporting the high relevance of the immune system in the development of this disease and suggesting that IgA vasculitis may be related to other class II vasculitides such as giant cell arteritis (GCA)^12^ or AAV^17^. The strongest signal mapped to the *HLA-DQA1/DQB1* region, which is in high LD with the *HLA-DRB1* gene. Consequently, the associated polymorphisms may be tagging a putative aetiologic variant at *HLA-DRB1*. Regarding polymorphic amino acid positions, none of the signals reached the genome-wide level of significance. Nevertheless, likewise rheumatoid arthritis^13^ and GCA^12^, the HLA-DRB1 positions 13 and 11 were amongst the strongest signals, which support the notion that IgA vasculitis may share immunopathogenic pathways with these conditions. On the other hand, after performing the conditional analysis on the HLA data, a potential signal that maps to *HLA-B* was observed, although it did not reach the genome-wide level of significance. This result could be indicating a potential effect of HLA class I in the pathogenesis of IgA vasculitis, as previously proposed^4^. In addition, no consistent associations with IgA vasculitis susceptibility were detected outside the HLA region, probably due to an insufficient statistical power to detect risk variants with a moderate effect.

Vasculitides constitute a heterogeneous group of diseases that often have overlapping clinical and pathological manifestations^18^. Nevertheless, differences between them in molecular terms have been described^7^. In this regard, the results derived from our study classify IgA vasculitis as a HLA class II condition linking it to GCA and AAV. Nonetheless, it is important to keep in mind that the number of cases recruited in our study was not high and replication was not carried out. Because of that, further confirmatory studies in independent populations should be performed to validate our data.

In summary, our results suggest that IgA vasculitis is an archetypal HLA class II disease.

## Electronic supplementary material

Supplementary information
