# Supplementary material for: A genome-wide association study suggests the HLA Class II region as the major susceptibility locus for IgA vasculitis
Source: Sci Rep. 2017 Jul 11;7:5088. doi: 10.1038/s41598-017-03915-2 (PMC5506002; doi:10.1038/s41598-017-03915-2)

A genome-wide association study suggests the HLA Class II region as the major susceptibility locus for IgA vasculitis

Raquel López-Mejías[1¥*], F. David Carmona[2,3¥], Santos Castañeda[4], Fernanda Genre[1], Sara Remuzgo-Martínez[1], Belén Sevilla-Perez[5], Norberto Ortego-Centeno[5], Javier Llorca[6], Begoña Ubilla[1], Verónica Mijares[1], Trinitario Pina[1], José A. Miranda-Filloy[7], Antonio Navas Parejo[8], Diego de Argila[9], Maximiliano Aragües[9], Esteban Rubio[10], Manuel León Luque[10], Juan María Blanco-Madrigal[11], Eva Galíndez-Aguirregoikoa[11], David Jayne[12], Ricardo Blanco[1§], Javier Martín[2§] and Miguel A. González-Gay[1,13,14§].

Supplementary Table S1. Main clinical and epidemiological features of a series of 285 patients with IgA vasculitis and 1,006 healthy controls.

Main characteristics	Patients<br>% (n/N)	Controls<br>% (n/N)
Male/ female	143/142	496/510
Children (age ≤20 years)/ adults (age >20 years)	231/54	0/1,006
Age at the onset of the disease (years, median [IQR])	7 [5-18]	-
Age at the study (years, median [IQR])	25 [14-37]	48 [27-62]
Duration of follow-up (years, median [IQR])	1.5 [1-4]	-
Palpable purpura and/or maculopapular rash	100 (285/285)	-
Arthralgia and/or arthritis	64.5 (184/285)	-
Gastrointestinal manifestations (if “a” and/or “b”)	60.7 (173/285)	-
a) Bowel angina	58.9 (168/285)	-
b) Gastrointestinal bleeding	18.6 (53/285)	-
Renal manifestations (if any of the following characteristics)	38.2 (109/285)	-
a) Hematuria	37.2 (106/285)	-
b) Proteinuria	30.5 (87/285)	-
c) Nephrotic syndrome	4.2 (12/285)	-
d) Renal sequelae (persistent renal involvement)*	7.3 (21/285)	-

IgA: Immunoglobulin-A; IQR: Interquartile range.

*At last follow-up.

Supplementary Table S2: Quality control procedures. Number of remained variants and samples after each filter are shown.

	Raw data	Cluster separation (< 0.4)	Low quality samples (mind < 0.05)	Low quality SNPs (geno < 0.02)	HWE (p < 0.001)	MAF (< 0.01)	IBD (> 0.4)	PCA (> 4 SD)	1KGPh3 imputation
# SNPs	306,670	306,009	306,009	288,395	288,152	240,286	240,286	240,286	2,581,927
# Patients with IgAV	308	308	286	286	286	286	286	285	285
# SNPs	306,670	305,956	305,956	283,544	282,161	231,652	231,652	231,652	2,185,351
# Controls	1,018	1,018	1,010	1,010	1,010	1,010	1,008	1,006	1,006

SNPs: single nucleotide polymorphisms; IgAV: IgA vasculitis; HWE: Hardy-Weinberg equilibrium; MAF: minor allele frequency; IBD: identity by descent; PCA: principal component analysis; SD: standard deviation from cluster centroid; 1KGPh3: 1000 genomes phase III panel.

Total number of SNPs after merging patients and controls: 1,909,910

Total number of individuals after merging patients and controls: 1,291

Supplementary Table S3. Overall statistical power of the study according to MAF and OR.

MAF	OR=1.2	OR=1.3	OR=1.4	OR=1.5	OR=1.7	OR=2.0
**0.05**	0.12	0.22	0.35	0.49	0.76	0.96
**0.10**	0.21	0.39	0.60	0.78	0.96	1.00
**0.15**	0.28	0.52	0.75	0.90	1.00	1.00
**0.20**	0.34	0.62	0.84	0.95	1.00	1.00
**0.30**	0.43	0.73	0.92	0.98	1.00	1.00
**0.40**	0.47	0.78	0.94	1.00	1.00	1.00
**0.50**	0.49	0.79	0.94	1.00	1.00	1.00

MAF: minor allele frequencies; OR: odds ratios.

Supplementary Table S4. Potential signals of association with IgA vasculitis susceptibility outside the HLA after imputation of GWAS data.

CHR	SNP	BP (GRCh37)	Reference	P	OR [CI 95%]	*Locus*	Position
2	rs12472112	43.314.451	T	3.11E-06	1.79 [1.40-2.28]	*LOC100506047*	Intronic
2	rs1002796	43.314.843	T	2.38E-06	1.79 [1.41-2.29]	*LOC100506047*	Intronic
2	rs1446468	164.963.486	T	6.20E-06	0.64 [0.53-0.78]	*FIGN-GRB14*	Intergenic
5	rs6883311	172.801.107	T	5.48E-06	1.62 [1.32-1.99]	*STC2 - LOC105377732*	Intergenic
10	rs11015915	19.013.396	A	9.03E-06	2.20 [1.56-3.12]	*LOC105376440*	Intronic
10	rs11015922	19.014.756	G	9.23E-06	2.20 [1.55-3.12]	*LOC105376440*	Intronic
10	rs11015929	19.015.486	G	9.23E-06	2.20 [1.55-3.12]	*LOC105376440*	Intronic
10	rs10826351	19.034.388	A	9.23E-06	2.20 [1.55-3.12]	*LOC105376440*	Intronic
10	rs12359075	19.053.551	G	9.75E-06	2.20 [1.55-3.12]	*LOC105376440*	Intronic
10	rs72781262	19.178.881	G	9.29E-06	2.18 [1.54-3.07]	*3' of LOC105376440*	Intergenic
11	rs2033836	96.562.777	T	4.03E-06	2.08 [1.53-2.85]	*3' of LOC105369443*	Intergenic
11	rs11212409	96.568.786	G	3.01E-06	1.91 [1.46-2.51]	*3' of LOC105369443*	Intergenic
11	rs10789640	96.569.259	G	2.49E-06	1.92 [1.46-2.51]	*3' of LOC105369443*	Intergenic
11	rs7945466	96.571.163	C	7.16E-06	1.86 [1.42-2.43]	*3' of LOC105369443*	Intergenic
11	rs2118983	96.571.547	A	2.64E-06	1.91 [1.46-2.50]	*3' of LOC105369443*	Intergenic
11	rs10890823	96.571.872	C	2.64E-06	1.91 [1.46-2.50]	*3' of LOC105369443*	Intergenic
11	rs7119456	96.572.887	A	5.55E-06	1.88 [1.43-2.46]	*3' of LOC105369443*	Intergenic

IgA: Immunoglobulin-A; HLA: Human leukocyte antigen; GWAS: genome-wide association study; CHR: chromosome; SNP: single nucleotide polymorphism; BP: base pair; OR: odds ratio; CI: confidence interval.

Supplementary Table S5. Signals associated with IgA vasculitis susceptibility at the genome-wide significance level-P<5E-08-after imputation of HLA data.

CHR	SNP	BP (GRCh36)	Reference	P	OR [CI 95%]
**6**	**rs9275224**	**32767856**	**A**	**5.74E-09**	**0.56 [0.46-0.68]**
6	rs6457617	32771829	C	1.56E-08	0.57 [0.47-0.69]
6	rs6457620	32771977	G	1.56E-08	0.57 [0.47-0.69]

IgA: Immunoglobulin-A; HLA: Human leukocyte antigen; CHR: chromosome; SNP: single nucleotide polymorphism; BP: base pair; OR: odds ratio; CI: confidence interval. Polymorphism highlighted **in bold** exhibit the most significant association.

Supplementary Table S6. Highest P-values of polymorphic amino acid positions obtained after imputation of the HLA data.

HLA molecule	Aminoacid position	Center codon position	Tested Alelles	X^2	P
DQB1	37	32740726	3	23.09505	9.66E-06
DQB1	55	32740672	3	23.17132	9.30E-06
DQB1	-10	32742295	3	23.28662	8.78E-06
DQB1	74	32740615	3	23.41474	8.23E-06
DRB1	96	32657590	4	28.00632	3.62E-06
DQB1	52	32740681	2	21.47537	3.58E-06
DQB1	47	32740696	2	21.47537	3.58E-06
DQB1	28	32740753	2	21.47537	3.58E-06
DQB1	46	32740699	2	21.47537	3.58E-06
DQB1	66	32740639	2	23.63098	1.17E-06
DQB1	67	32740636	2	23.63098	1.17E-06
DRB1	13	32660109	6	26.65069	6.67E-05
DRB1	26	32660070	3	20.71187	3.18E-05
DQB1	30	32740747	3	21.72689	1.91E-05
DRB1	11	32660115	5	27.12367	1.88E-05
DQB1	71	32740624	4	25.13019	1.45E-05
DQB1	224	32737107	2	19.04256	1.28E-05
DRB1	73	32659929	2	19.15778	1.20E-05

HLA: Human leukocyte antigen

Supplementary Fig. Principal component analysis for the first three principal components for each individual.

Patients with IgAV are shown in blue. Healthy controls are represented in red. Those deviating more than 4 standard deviations from the cluster centroids were discarded from further analysis.

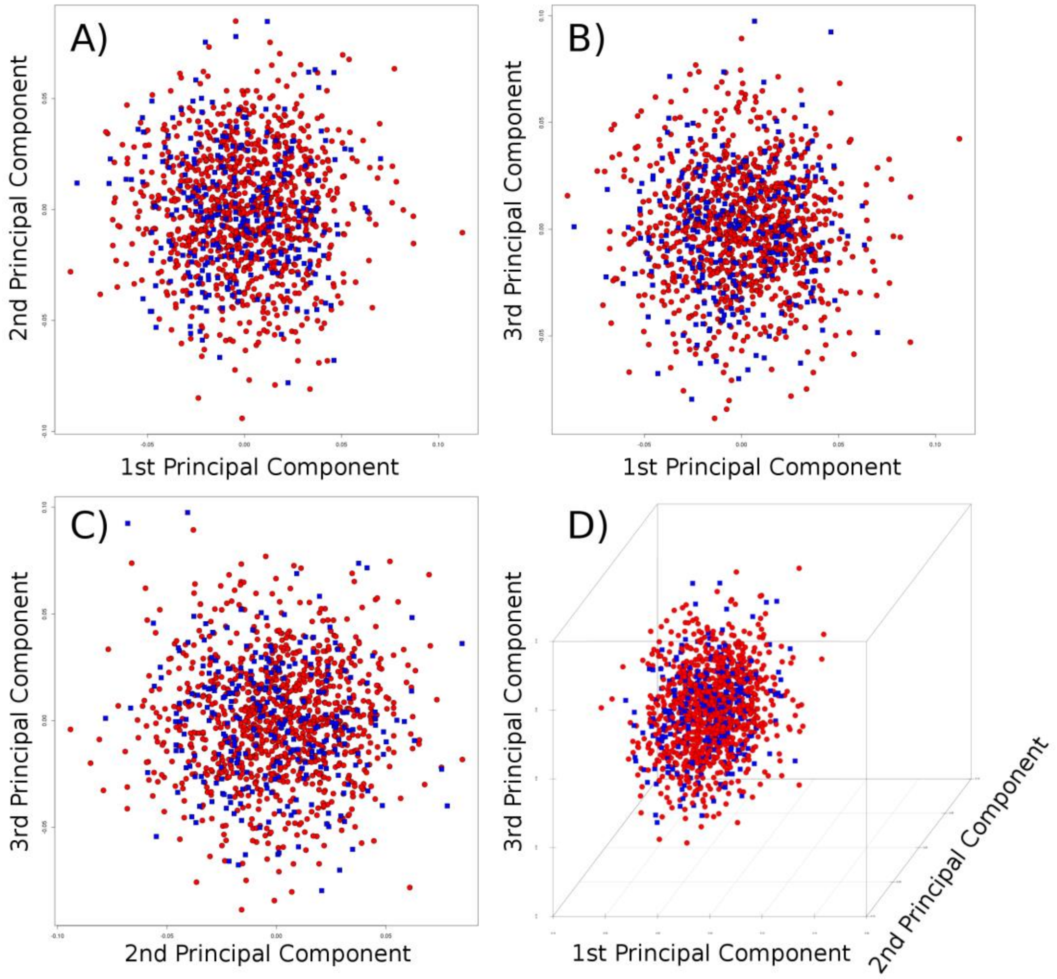

Supplement: Supplementary file 1 — Supplementary information [file 41598_2017_3915_MOESM1_ESM.pdf]
